# Examination of the Vitreolenticular Interface in Relation to Uneventful Phacoemulsification over One-Year Postoperative Period

**DOI:** 10.3390/jcm13113219

**Published:** 2024-05-30

**Authors:** Ágnes Elekes, Gábor Németh, Dóra Lauter, Márton Edelmayer, Zsófia Rupnik, Péter Vámosi

**Affiliations:** 1 Department of Ophthalmology, Péterfy Sándor Hospital, 1076 Budapest, Hungarymartonedelmayer@gmail.com (M.E.); zsofia.rupnik@gmail.com (Z.R.); vamosipeter@freemail.hu (P.V.); 2 Faculty of Health Sciences, Institute of Applied Health Sciences, University of Miskolc, 3515 Miskolc, Hungary; 3 Department of Ophthalmology, University of Debrecen Faculty of Medicine, 4032 Debrecen, Hungary

**Keywords:** anterior hyaloid membrane, Berger’s space, anterior segment optical coherence tomography, anterior vitreous detachment

## Abstract

**Background**: Swept-source anterior segment optical coherence tomography (SS-AS-OCT) is a suitable examination for the vitreolenticular interface. **Methods**: In a prospective study using Anterion (Heidelberg Engineering, Heidelberg, Germany), 102 eyes of 102 patients were examined in pupil dilation, preoperatively and 6 times over 1-year follow-up. Preoperatively anterior hyaloid membrane (AHM) visibility was determined with Imaging App with high reliability. Postoperatively capsular bag–AHM distance was measured on six points by using Metrics App. **Results**: The AHM was visible in 18.6% preoperatively and postoperatively as well (Group 1), 49% of the preoperatively adherent AHMs became visible (Group 2A), 32.4% remained attached (Group 2B). Group 1: the average deepest point on the first day was 782.5 ± 324.1 microns, and it significantly differed from the later follow-up values. Group 2A: the average deepest value was 184.1 ± 220.1 microns, and there was no statistically significant difference between the postoperative visit values. The difference between the groups was statistically significant at every location and at each time point. **Conclusions**: AS-SS-OCT can be used to check BS both preoperatively (with limitations) and postoperatively.

## 1. Introduction

Berger’s space (BS), named after Emil Berger, refers to the potential compartment between the posterior surface of the lens and the vitreous humor. Wieger, a colleague of Berger, identified the hyalocapsular ligament (also known as Wieger’s ligament), forming a ring 8 to 9 mm in diameter 1 mm from the periphery of the lens, which binds the vitreous to the posterior lens surface [1]. Morsman provided an overview of the biomicroscopic features of the retrolental space in 1929, underscoring its clinical significance in anterior and posterior segment inflammations [2]. Tolentino et al. reported blood deposits on the posterior lens capsule in cases of proliferative diabetic retinopathy, highlighting another clinical relevance of BS [3]. In the era of intracapsular cataract extraction it was a crucial question how the surgeon can gently disrupt the zonular fibers and the Wieger’s ligament and remove the lens without causing vitreous loss [4]. When extracapsular cataract extraction (ECCE) became a general practice, preservation of the lens capsule and the anterior hyaloid membrane (AHM) was one of the main goals. However, in special cases of ECCE, management of BS was necessary. In membranous capsular changes Weidle et al. punctiformly opened the posterior capsule and filled the retrolental space with sodium hyaluronate for widening this before primary posterior capsulectomy [5]. Tassignon and Dhubhghaill visualized the BS first with real-time intraoperative optical coherence tomography (iOCT) during phacoemulsification [6], while Mares et al. documented the BS using an anterior segment optical coherence tomograph (AS-OCT) in a clinical setting [7]. Lin et al. investigated the effect of phacoemulsification on the BS by slit-lamp biomicroscopy and with AS-OCT before and one month after surgery [8]. Using swept-source AS-OCT (SS-AS-OCT) Zhang et al. concluded that after phacoemulsification, locations with a much wider BS are predominantly located in the opposite direction to the main corneal incisions [9]. Mori et al. investigated AHM with SS-AS-OCT and concluded that the patients with visible AHM were significantly older than those without visible AHM [10]. In our study, we investigated the presence and development of BS and its change in the perioperative and late postoperative period related to uneventful phacoemulsification based on measurements of SS-AS-OCT. To the best of our knowledge, this is the first prospective report documenting BS evolution over a one-year period.

## 2. Materials and Methods

In this prospective non-randomized interventional study, we assessed the evolution of the retrolental space before and after cataract surgery using SS-AS-OCT. The study was conducted with approval from the local ethics committee. The study was conducted in accordance with the Declarations of Helsinki.

### 2.1. Patients

Between 2021 July and 2023 July, we examined 102 eyes of 102 patients: preoperatively, on 1 day, 1 week, 1 month, 3 months, 6 months, and 1 year follow-up. At each check-up, visual acuity, intraocular pressure, pupil dilation and slit-lamp examination with fundus examination were performed. Preoperatively and on each follow-up, SS-AS-OCT in pupil dilation were performed with the Anterion device (Heidelberg Engineering, Heidelberg, Germany).

### 2.2. Image Acquisition

Eyes with cornea, iris, or lens malformations, phacodonesis, poorly dilated pupils, or eyes previously undergone any eye surgery were excluded from the study. Out of the initial 120 eyes, in 1 case posterior capsular rupture occurred, and 17 patients did not come for regular check-ups; these eyes were also excluded from the study.

**Figure 1:**
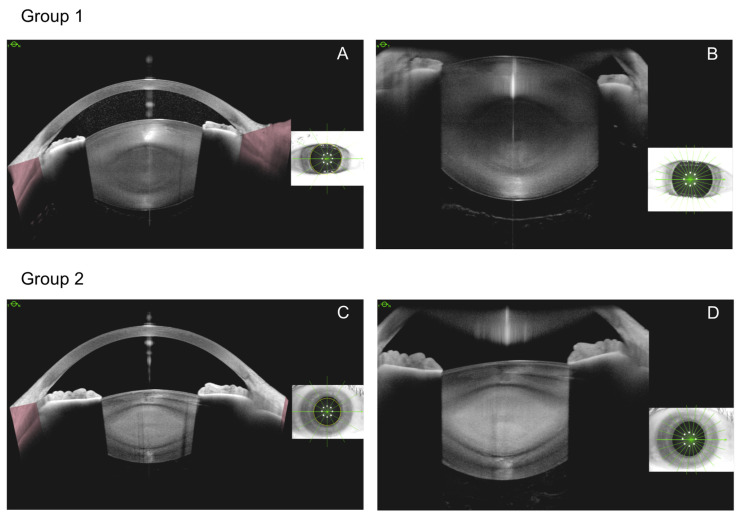
Imaging methods, preoperative images (**A**–**D**). Group 1, same eye—Metrics App (**A**) with invisible AHM, and Imaging App (**B**) with visible AHM (yellow arrow). Group 2 Metrics App (**C**), Imaging App (**D**), same eye with invisible AHM.

Preoperatively, we performed intraocular lens power calculation with the Cataract App of Anterion device, and we also captured eyes using the Metrics App. The Metrics App automatic mode creates multiple radial B-scans up to 16.5 mm in length and 14.0 mm depth using 1300 nm wavelength. The images taken clearly depict the cornea, anterior chamber, angle of the chamber, iris, and lens, but the clear visibility of retrolental space in phakic eyes is limited. For that reason, the depth of BS was not measured. The presence or absence of AHM was checked in the Imaging App. Turning off the Imaging App automatic mode, in manual settings, by pushing the device closer to the eyes it was possible to take deep and clear images of retrolental space. While conducting our study, Zicarelli et al. used a similar method to examine the anterior vitreous in eyes with vitritis [11]. The Imaging App mode was only used for preoperative examination as an additional certainty inspection, but correct measurements in this mode were not possible. By choosing the most appropriate dense pattern in Imaging App manual mode, we made sure if the hyperreflective line visible in the Metrics mode is not just a vitreous fiber, but actually the AHM. Figure 1 shows the preoperative imaging method.

Postoperatively, the distances measured were given in microns, for which we only used the Metrics App automatic mode in all the postoperative follow-ups. Along the intraocular lens (IOL), in the area not shaded by the iris, AHM was clearly visible and suitable for measurements.

**Figure 2:**
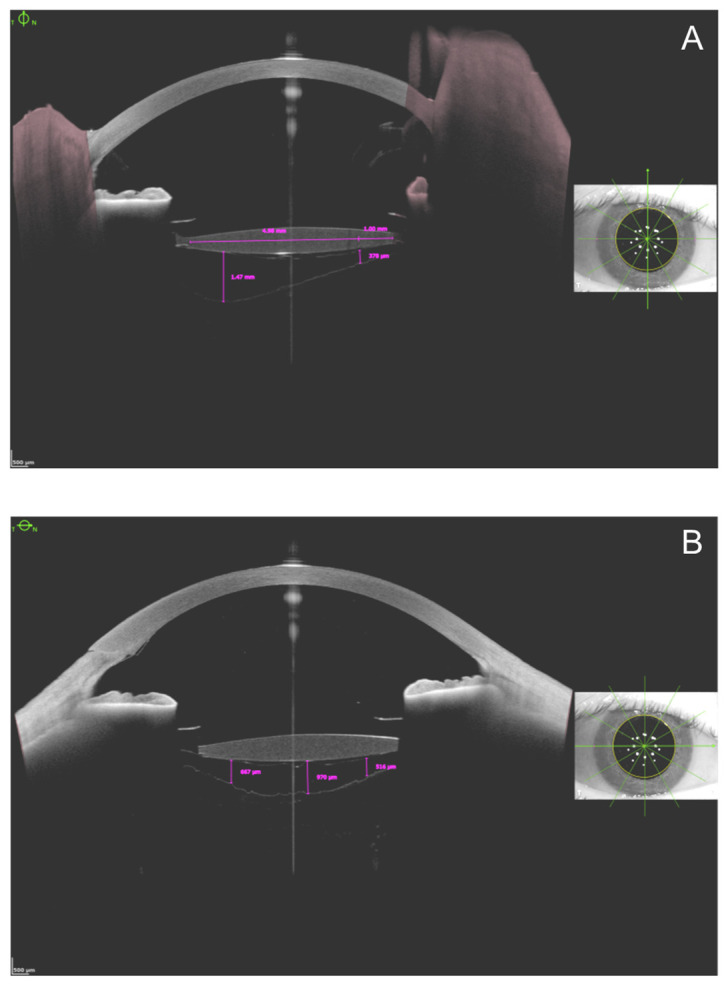
Postoperative measurements on vertical (**A**) and horizontal (**B**) images.

For the measurements, B-scans of the 90–270 degrees vertical section and the 0–180 degrees horizontal sections were used. On each scan we measured the distance between the posterior capsule and the AHM on three points. As an agreement, for measurements we picked two surely visible points both sides of the IOL: 1 mm centrally from the optic edge, respectively. We measured the BS width at the deepest point as well (Figure 2).

We found the deepest point as follows: the 6 mm axis of the optic of the IOL was drawn, connecting the central edges of the haptics. This axis was shifted parallel and removed from its original location. The farthest point where the straight line still touched the anterior hyaloid membrane was called the deepest point. The distance was given by a perpendicular line to the parallel of the axis. When the anterior hyaloid detachment was uniform, or the posterior capsule was uneven or loose, the distance was measured at several deep points, as many times as it was necessary.

In the images, the posterior capsule is hyperreflective and appears as a continuous line following the shape of the IOL. The AHM is a thinner, less hyperreflective, slightly irregular line. The images were checked by an experienced examiner. In a separate session, measurements were completed by another experienced examiner. Accordingly, the nasal and temporal, the lower and upper 1 mm depths and the deepest point were determined on each follow-up.

### 2.3. Surgical Procedure

The operations were performed by a single surgeon. A 2.7 mm clear corneal incision was placed in the temporal 0–180 degrees, followed by uneventful phacoemulsification and implantation of a one-piece IOL into the capsular bag.

### 2.4. Statistical Analysis

In the case of quantitative variables, the valid N, mean, standard deviation, minimum, 25th percentile, median, 75th percentile and maximum values were given, while in the case of qualitative variables, the number of cases and the percentages were used.

For qualitative variables, Fisher’s test or Pearson’s chi-square test was used.

To select the right statistical test, we checked the normality of the variables using Shapiro–Wilk test and the equality of error variances by Levene’s test. The Mann–Whitney U-test was applied to test the differences between the individual groups when the Shapiro–Wilk test was significant (*p* < 0.05) and independent *t*-test (two groups comparison) or one-way ANOVA with Bonferroni post-hoc test (three groups comparison) was performed in case of variables where the Shapiro–Wilk test did not detect significant deviation from normal distribution.

To test the time differences between the individual groups, we performed the Friedman test in the case of variables where the Shapiro–Wilk test displayed significant deviations from normal distribution and Levene’s test rejected the null hypothesis that the error variance of variable is equal across tested groups.

A correlation analysis was performed to compare the groups (Spearman’s correlation). In general, significance level was set up at *p* < 0.05. In cases where a *p*-value adjustment was necessary due to multiple hypotheses, the Bonferroni method was used to determine the significance level.

To test the intraobserver differences of the measurements, the same investigator measured the distances between the bag and AHM three times in a separate group of 20 eyes. Repeatability was described by the intra-observer standard deviation (Sw), the test-retest variability (coefficient of repeatability, CoR) and the coefficient of variation (CoV). CoR was calculated as Sw times 2.77. CoV was calculated as the ratio of Sw to the mean of the measurements expressed as a percentage. All calculations were based on data from the six measurement points. Sw was between 1.95 and 6.31 microns, CoR was between 5.41 and 17.49 microns and CoV was between 1.61 and 2.83%.

Similarly and separately for Group 1, the same investigator measured the distances between the bag and AHM 3 times for 18 eyes. Sw was between 15.68 and 32.87 microns, CoR was between 43.45 and 91.06 microns and CoV was between 2.9 and 5.4%. ICCs were between 0.968 and 0.996.

All statistical tests were performed using IBM SPSS version 25.

## 3. Results

A total of 102 eyes were included in the database. The examined eyes were divided into two groups. Group 1 includes eyes with visible BS preoperatively (N = 19) while Group 2 includes eyes without visible BS preoperatively (N = 83). Group 2 was divided into two subgroups depending on the postoperative outcome: Group 2A (N = 50) included eyes in which BS was detectable at any time during the postoperative follow-up, but at least at one follow-up. Group 2B included eyes of Group 2 without detectable BS at any postoperative follow-up (N = 33).

Preoperatively, AHM was visible with the Imaging App in 19 cases, while on the images of the Metrics App only 8 cases of AHM detachment were detected. Therefore, more than half of the cases would have been lost without the control method we recommend.

Intraobserver differences were checked to validate the reliability of the measurements in two different sittings: the same investigator measured the distances between the bag and AHM 3 times in a group of 20 eyes (Sw was between 1.95 and 6.31 microns, CoR was between 5.41 and 17.49 microns and CoV was between 1.61 and 2.83%.), then, separately, the measurements were carried out for eyes of Group 1(Sw was between 15.68 and 32.87 microns, CoR was between 43.45 and 91.06 microns and CoV was between 2.9 and 5.4%. ICCs were between 0.968 and 0.996.). Both series of calculations showed excellent reliability of measurements.

Table 1 shows the baseline distribution of age, gender, axial length (AL), anterior chamber depth (ACD) and lens thickness (LT), summarizing the statistical correlations discussed in detail in the following text.

A total of 26 (25.5%) men and 76 (74.5%) women were included in the study. In Group 1: 3 (15.8%) men and 16 (84.2%) women, in Group 2: 23 (27.7%) men and 60 (72.3%) women. There was no statistically significant correlation between groups and gender.

The average age was 71.68 ± 6.75 years in Group 1 and 68.39 ± 9.94 years in Group 2, with no statistically significant difference. The average age in Group 2A was 71.30 ± 8.945 years, while in Group 2B it was 63.97 ± 9.87 years. A statistically significant difference was observed between the three groups (ANOVA, F: 7.785, *p* = 0.001). During the pairwise comparison, the difference between 1 and 2B (Bonferroni adjusted *p*-value: 0.01) and 2A and 2B (Bonferroni adjusted *p*-value: 0.001) was statistically significant (Table 1).

In Group 1, the average axial length was 22.98 ± 0.77 mm, while in Group 2 it was 24.38 ± 2.22 mm. The difference between the two groups was statistically significant (independent *t*-test; *t* = −4.629; *p* < 0.001). In Group 2A, the average axial length was 23.79 ± 1.67 mm, while in Group 2B it was 25.26 ± 2.66 mm. A statistically significant difference was observed between the three groups (ANOVA, F: 9.670, *p* < 0.001). During the pairwise comparison, the difference between 1 and 2B (Bonferroni adjusted *p*-value < 0.001) and 2A and 2B (Bonferroni adjusted *p*-value: 0.003) was statistically significant (Table 1).

The ACD was 258.8 ± 43.95 in Group 1, 270.0 ± 40.69 in Group 2A, and 276.6 ± 39.38 in Group 2B. According to the results of the ANOVA test, there was no significant difference between the groups (*p* = 0.326) (Table 1).

The LT in Group 1 was 4.617 ± 0.454, in Group 2A 4.637 ± 0.469 and in Group 2B 4.753 ± 0.427. According to the results of the ANOVA test, there was no significant difference between the groups (*p* = 0.448) (Table 1).

**Figure 3:**
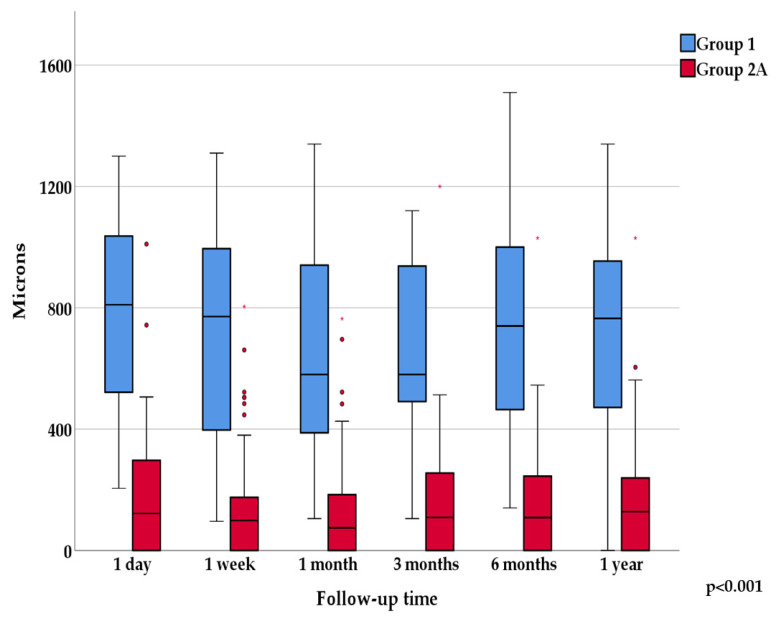
Shows the mean values of the deepest points over one-year follow-up on horizontal AS-OCT sections.

On horizontal cross-sectional images on the first postoperative day, the deepest point was located in the nasal region in most cases in Group 1 (68.4%), while in Group 2A it was located centrally (60.0%). The difference between the groups was statistically significant (*p* = 0.001). The average depth of the deepest point on horizontal meridian at the first day follow-up was 782.5 ± 324.1 microns (N = 19) in Group 1 and 184.1 ± 220.1 microns (N = 50) in Group 2A. There was one case with less than 220 microns BS in Group 1, and also one with more than 700 microns deepest depth in Group 2. The one-year, six occasion follow-up values are shown in Figure 3.

Examining the progression over time, the change in average depths in Group 1 was statistically significant (*p* = 0.031), while in Group 2A there was no statistically significant change in depth over one year.

**Figure 4:**
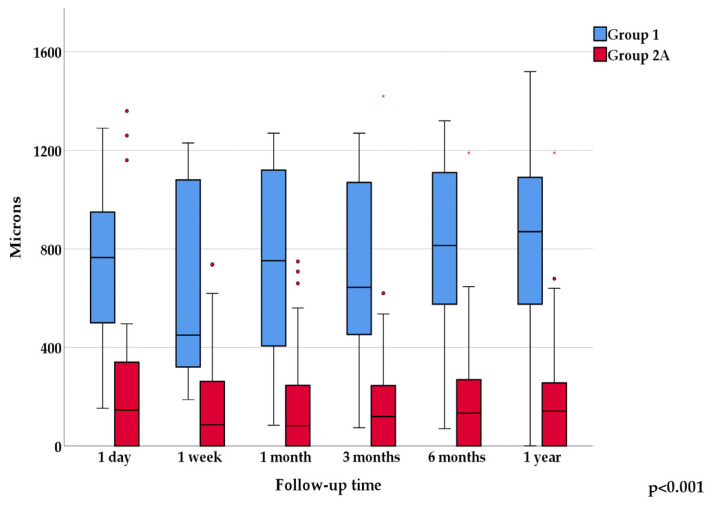
Shows the mean values of the deepest points over one-year follow-up on vertical AS-OCT sections.

On vertical cross-sectional image on postoperative first day, the deepest point was most often located in the inferior region in Group 1 (78.9%), while in Group 2A it was located in the central region (50.0%). The difference between the groups was statistically significant (*p* = 0.004). When examined over time, there was no statistically significant change regarding the location in either of the two groups. The average depth of the deepest point on vertical meridian on postoperative day one was 711.3 ± 332.5 microns in Group 1 and 231.7 ± 310.4 microns in Group 2A. The one-year, six occasion follow-up values are shown in Figure 4.

Examining the progression over one-year, six occasion follow up, the change in average depths showed no statistically significant difference neither in Group 1, nor in Group 2A (Friedman test, *p* = 0.031); whereas there was a significant difference between the two groups at each time point (Mann–Whitney test, Bonferroni adjusted *p* < 0.008).

**Figure 5:**
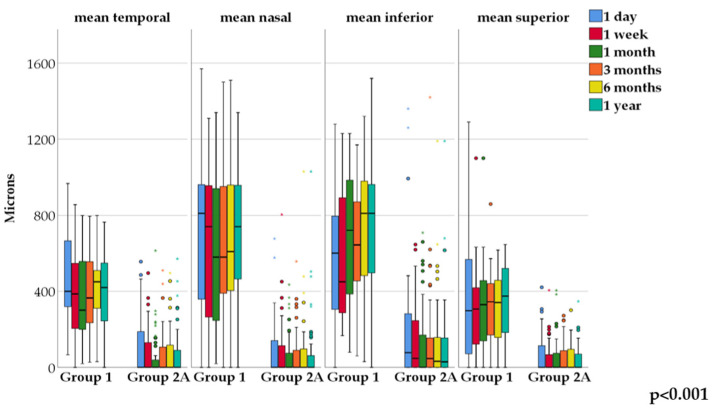
Change of mean values over one year in Group 1 and Group 2A.

In Group 1, the Friedman test showed no statistically significant changes in the nasal, temporal, superior or inferior areas during the one-year follow-up period compared to the first postoperative day. In Group 2A, there was a significant change from baseline in the nasal and temporal regions on the first postoperative day (*p* = 0.002 and *p* = 0.008, respectively), while there was no significant change in the inferior and superior regions. Mann–Whitney test showed a significant difference between Group 1 and 2A at each time point in all four areas—nasal, temporal, inferior and superior (*p* < 0.001) (Figure 5).

Spearman’s correlation test showed a statistically significant correlation between inferior and superior areas only in Group 2A at each time point (*p* < 0.05). Spearman’s correlation test showed a statistically significant correlation between nasal and temporal areas at each time point in Group 2A and only on first week follow-up in Group 1 (*p* < 0.05).

On horizontal cross-sectional images, on first day, the deepest point was predominantly in the nasal area in Group 1, whereas in Group 2A it was in the central area. The difference between the two groups was statistically significant (*p* < 0.001). Examining the individual groups over time, there were no statistically significant changes. On vertical cross-sectional images, the deepest point was in the inferior region in Group 1, and centrally in Group 2A. The difference between the two groups was statistically significant (*p* = 0.001). Examining the individual groups over time, there were no statistically significant differences.

## 4. Discussion

Theoretically many factors can influence the visibility of AHM on anterior segment OCT, i.e., the presence or absence of BS preoperatively. There are circumstances of the measurement, type and density of cataract, age, axial lengths. In previous studies BS was identified with AS-OCT in different percentages before surgery, BS was identified at a different percentage before surgery. Zang et al. found 0.9% [9], Lin et al. 3.4% [8], Mori et al. 8.1% of cases [10], while in our study, AHM was visible in 18.6%. More recently, Lin et al. found 2.5% of cases AVD (anterior vitreous detachment) preoperatively using iOCT [12]. The age of cataract patients was comparable in the different studies: 66.0, 65.0, 70.1, and 69.0 years, respectively. However, the circumstances of the measurements were not the same. Lin’s AS-OCT was unspecified [8], and Mori et al. measured with CASIA2 (Tomey; Nagoya, Japan) deep-range SS-AS-OCT [10]. Zang et al. [9], as well as our team, used Anterion SS-AS-OCT. However, Zang et al. captured images with Metrics App automatic mode. Preoperatively, we used the Imaging App manual settings to take deep and clear images of retrolental space. Another difference is that Lin et al. did not indicate the status of the pupil [8], Zang et al. measured without pupillary dilation [9], while Mori et al. [10] and we dilated the pupil before examination. Theoretically it is possible that the opaque lens might have influenced the visibility of AHM. Mori et al. found that detection rate of AHM was not influenced by the LOCS III gradings of cataract [10]. According to Mori et al., patients with visible AHM were significantly older than those without visible AHM [10]. In our study the difference was not statistically significant, but we found a tendency towards older age. The role of axial length is controversial. Mori et al. reported that eyes with visible AHM had significantly greater axial length. Based on our observation, AHM visibility is not related to the elongated axial length; in fact, preoperatively adherent AHM was more common in longer eyes (*p* < 0.001). The considerable disparity of detectable BS in different populations before cataract surgery can be related to different races as well. In agreement with the literature data, we found that there are several factors relevant for a preoperatively detectable BS, as measurement method, which enables reliable deep penetration, pupil dilatation and age.

It is well known that phacoemulsification has a significant impact on BS [6,8,9,10,12,13,14,15]. In certain cases, using iOCT an open BS with some small floating hyperreflective materials is detectable during phacoemulsification [6,8,12,13,14], or a circular rupture of the AHM can occur [12]. Anisimova et al. found the BS to be open in 82% of the cases in first week after surgery [14]. Vael et al. reported AVD in 63% of cases following bag-in-the-lens cataract surgery [15]. Lin et al. identified the BS in 27.7% [8], while Zhang et al. found it in 19.7% in one month after phacoemulsification [9]. 

Our aim was to observe the vitreolenticular surface in the case of cataractous but otherwise healthy eyes, and to follow the evolution of the anterior vitreous surface after an uneventful phacoemulsification for one year. Until now, it was not clear how the vitreolenticular interface changes in the late postoperative period. We found a striking difference in postoperative pictures during the one-year follow-up between the two examined groups. The average depth value of the deepest AHM detachment was around 650–750 microns in Group 1, and 140–180 microns in Group 2A during a one-year postop period. The difference between the two groups was statistically significant at the largest capsular bag–AHM distance, and at the four peripheral locations at each time point. For Group 1, the deepest point on the horizontal cross-sectional images was in the nasal region in most cases, gradually decreasing from day 1 to 3 months follow-up and then increasing again at the 1-year visit. At every follow-up and every measurement point, there was a great AHM–capsular bag distance (more than 350 microns), except for one eye, where the BS became virtual at the one-year follow-up visit. In the horizontal cross-sectional images, the difference was significant between the one-day baseline visit and all subsequent follow-ups.

As originally defined, the BS is a space located between the posterior capsule of the lens and the anterior membrane of the vitreous, structures that adhere in a circular manner by means of the Wieger’s ligament, for which the outer limit is defined by Egger’s line. The area bounded by the ligament is called BS. Thus, in cases where it is suspected that the Wieger’s ligament is torn or detached, so that it does not keep the AHM in its original anatomical position, it is no longer correct to use the term BS. Rather, AHM detachment or AVD is a more appropriate term [12,14,16].

In Group 2A, horizontal cross-sectional images’ deepest values measured were centrally located. The first-day values gradually decreased until the one-month follow-up, and then slightly increased for the one-year visit. However, this was only a trend, but it was not significant. A similar trend was observed in the vertical cross-sectional images. In Group 2A, similar values were observed for only one eye as in Group 1, with the deepest point varying between 1010 and 647 microns over the follow-up period. 

In Group 1, vertical images, the largest AHM detachment region, which was in 78.9% the lower region, deepens in the early postoperative period, then the values decrease, and it deepens again at the one-year visit. The vertical values of Group 2A are much smaller, and it follows a path corresponding to the change of the horizontal section over time.

Based on the measured values and the cross-sectional images, the dynamics of the anterior vitreous body become clear during the first postoperative year.

However, we can also see that this happens slightly differently for the two groups. The difference is because the preoperatively detached AHM cannot reorganize in the immediate vicinity of the lens capsule. We strongly believe this phenomenon depends on the adhesion or detachment of the supporting ligament of AHM known as Wieger’s ligament. Wieger’s ligament cannot be visualized on OCT due to its peripheral location and iris coverage and due to its thin, fine structure, which adheres to the surrounding surfaces. Thus, we infer its position relative to the lens capsule from the aspect of the retrolental space that can be captured.

The typical location of the deepest point in Group 1 was in the nasal area on the horizontal cross-sectional images and in the lower area on the vertical cross-sectional images. The preoperative images were primarily suitable for depicting the visible BS in the center of the lens because the increased diameter of the cataractous lens interfered with detailed imaging. Therefore, we do not know whether the AHM has already detached in the nasal region and the lower region before surgery or not. However, the identical course of the Group 1 cases still suggests that phacoemulsification may cause further non-repairable damage to the weakened ligament. The position of the deepest points may indicate the direction of fluid flow in the retrolental area.

This hypothesis is supported by the evolution of the eyes of Group 2A over time as well. No preop AHM detachment was seen in Group 2A. In most cases, postoperatively, we can see a centrally opened small BS, and its average values are three times less than the group 1 values, or the space remained virtual (Group 2B). If the peripheral invisible supporting elements are preoperatively intact, the spatial rearrangement caused by phacoemulsification is less. 

The space left after the relatively large lens, behind the relatively narrow IOL, does not have a final morphology on the first post-op day and it does not even settle for the first month check-up. However, in Group 2A we found an AHM detachment of more than 700 microns in one eye, which is associated with the partial or total detachment of Wieger’s ligament caused by phacoemulsification. Of course, this theory must be supported by further histopathological examinations. 

Spearman’s correlation test showed statistically significant correlation of inferior and superior area values at each time point in Group 2A (*p* < 0.05) and a statistically significant correlation of nasal and temporal area values at each time point in Group 2A. This correlation was found only on first week follow-up in Group 1 (*p* < 0.05). The vitreous is not a rigid anatomical structure. In the early postoperative period, in Group 1 and Group 2A, there is a residual amount of fluid between the capsular bag and the AHM, which flows out from there in the late postoperative period. In Group 2A, the slow symmetric rearrangement shows that the fine structures show a certain degree of functionality.

In our study, the preoperative and also the postoperative AHM detachment was associated with eye length. In Group 1, the average axial length was in the normal range, being significantly shorter than the ones in Group 2. We presented in our database 26 eyes with higher axial length (above 26.0 mm, range: 26.02–33.02 mm); they all were found to be in Group 2, meaning we did not see the AHM to be detached preoperatively at any of these particular patients. Moreover, we found the eyes of Group 2B to be significantly longer than the ones of Group 2A. Therefore, AVD also shows a correlation with postoperative axial length. The preoperatively adherent AHM was not detached in the longer axial length subgroup, and it was found to be detached in the relatively shorter one. Keeping in mind that the posterior vitreous detachment (PVD) occurs at an early age in highly myopic eyes [17], we could assume a similar runoff for AHM. The average age of the highly myopic patients was 54. More than half of them did not even develop an AVD in one year after surgery, but two of the highly myopic eyes presented a postoperative pattern similar to the ones seen in Group 1: AHM stabilized in a greater distance from the posterior lenticular surface.

Therefore, multiple explanations come into view. The authors do not insist on the PVD-AVD formation parallel completely. We hypothesize that the aging processes in the anterior vitreous and the posterior vitreous are not the same. It is well known that in case of PVD liquefaction of the vitreous body, weakening of adhesion between the vitreous posterior cortex and the internal limiting lamina occur, which is more prevalent in older patients and in myopic eyes [18]. We consider posterior vitreous liquefaction as a possible cause of anterior vitreous adhesion to the lens capsule. Different tissue elements are involved in the vitreolenticular surface compared to the vitreoretinal surface. Thus, its pathology may also differ. On the other hand, in the process of axial elongation, the sclera undergoes significant remodeling. Its thickness decreases with a longer axial length, most markedly at the posterior pole, and least markedly at the ora serrata and anterior to the ora serrata [19]. Therefore, the load puts less strain on the vitreolenticular surface, making it less probable an AVD to be formed. Twenty-six eyes (25.49% of all examined eyes) are not the best representative sample size, and it needs further investigation.

Knowing of BS before cataract surgery also can help us to occasionally consider performing a primary posterior capsulorhexis. In view of the above, it is possible that detection of BS may soon become a routine part of the examination before and after cataract surgery.

Based on the literature and our current data, we can conclude that the vitreolenticular interface can go through an evolution. Before cataract surgery with AS-OCT [8,9,10] or with iOCT [14], AHM detachment can be observed in certain cases (18,6% in our study), but in most cases, the AHM is not visible, which means that it is attached to the lens capsule. Surgical [6,8,9,10,12,14,15] or other trauma [13,20,21,22,23,24] can stimulate the AHM detachment and in numerous cases a newly developed BS can be detected or a pre-existing BS can permanently enlarge. 

Our study has some weaknesses. We used the Imaging App of the Anterion AS-SS-OCT to check for the presence or absence of BS during preoperative assessment, but comparable measurements in this mode were not possible. We were able to detect the correct position of the AHM postoperatively, but we were unable to detect the Wieger’s ligament itself. Therefore, the status of the ligament was determined by deduction. The surgical parameters of the phacoemulsification have not been discussed in this manuscript and will be interpreted in another study.

## 5. Conclusions

In summary, this is the first prospective report documenting the evolution of BS and AHM over one year following phacoemulsification. Using SS-AS-OCT, we found a visible AHM in 18.6% preoperatively and an opened BS in 49% postoperatively, while in 32.4% of cases the AHM remained attached to the posterior capsule during the whole postoperative period. In Group 1, the average BS depth was around 750 microns in various locations, and the one-day postop depth differed significantly from the controls in the horizontal cross-sectional images, which means that the stabilization of the AHM is slower in this group. In Group 2A, the average BS depth was around 180 microns, and there was not a statistically significant difference between the postoperative visits. The preoperatively visible AHM was not associated with longer axial length, while the postoperatively opened BS was associated with the older age of patient. 

## Figures and Tables

**Table 1:** Distribution of age, gender, axial length (AL), anterior chamber depth (ACD) and lens thickness (LT) mean values.

	1 (N = 19)	2a (N = 50)	2b (N = 33)	*p*-Value
Gender				
male (n, %)	3, 15.8%	14, 28.0%	9, 27.3%	0.559
female (n, %)	16, 84.2%	36, 72.0%	24, 72.7%	
Age in years (mean ± SD)	71.7 ± 6.76	71.3 ± 8.94	64.0 ± 9.87	<0.001
Axial length (mean ± SD)	22.98 ± 0.773	23.79 ± 1.671	25.26 ± 2.657	≤0.003
Anterior chamber depth (mean ± SD)	258.8 ± 43.95	270.0 ± 40.69	276.6 ± 39.38	0.326
Lens thickness (mean ± SD)	4.617 ± 0.454	4.637 ± 0.469	4.753 ± 0.427	0.448

## Data Availability

Data will be available on request from the corresponding author.

## References

[1] Wieger G. (1883). Über den Canalis Petiti und ein ‘Ligamentum Hyaloideocapsulare’. Inaugural Dissertation.

[2] Morsman L.W. (1929). The retrolental space. Arch. Ophthalmol..

[3] Tolentino F.I., Lee P.F., Schepens C.L. (1966). Biomicroscopic study of vitreous cavity in diabetic retinopathy. Arch. Ophthalmol..

[4] Mortada A. (1971). Hyaloideocapsular ligament of Wieger and vitreous loss in the course of intracapsular lens extraction. Br. J. Ophthalmol..

[5] Weidle E.G., Lisch W., Thiel H.J. (1986). Management of the opacified posterior lens capsule: An excision technique for membranous changes. Ophthalmic Surg..

[6] Tassignon M.-J., Dhubhghaill S.N. (2016). Real-time intraoperative optical coherence tomography imaging confirms older concepts about the Berger space. Ophthalmic. Res..

[7] Mares V., Nehemy M.B., Salomao D.R., Goddard S., Tesmer J., Pulido J.S. (2020). Multimodal imaging and histopathological evaluation of Berger’s space. Ocul. Oncol. Pathol..

[8] Lin W.L., Geng W.J., Ji M., Li P.F., Luo L.W., Guan H.J. (2022). Effect of phacoemulsification on Berger space. Zhonghua Yan Ke Za Zhi.

[9] Zhang Z., Yao J., Chang S., Kanclerz P., Khoramnia R., Deng M., Wang X. (2022). Incidence and risk factors for Berger’s space development after uneventful cataract surgery: Evidence from swept-source optical coherence tomography. J. Clin. Med..

[10] Mori H., Ueno Y., Fukuda S., Oshika T. (2022). Detection of anterior hyaloid membrane detachment using deep-range anterior segment optical coherence tomography. J. Clin. Med..

[11] Zicarelli T., Staurenghi G., Invernizi A. (2023). Anterior segment optical coherence tomography (AS-OCT) visualization of anterior vitritis. Ocul. Immunol. Inflamm..

[12] Lin W., Luo J., Li P., Ji M., Guan H. (2023). Anterior vitreous detachment and retrolental material during cataract surgery: Incidence and risk factors, with pathological evidence. J. Cataract Refract. Surg..

[13] Naumann G.O.H. (1999). Correspondence. Photographs of intralenticular hemorrhage following blunt ocular trauma. Arch. Ophthalmol..

[14] Anisimova N.S., Arbisser L.B., Shilova N.F., Melnik M.A., Belodedova A.V., Knyazer B., Malyugin B.E. (2020). Anterior vitreous detachment: Risk factor for intraoperative complications during phacoemulsification. J. Cataract Refract. Surg..

[15] Vael A., Os L.V., Melis K., Tassignon M.-J. (2022). Evaluation of the vitreolenticular interface with intraoperative OCT. J. Cataract Refract. Surg..

[16] Santos-Bueso E. (2019). Berger’s space. Arch. Soc. Esp. Oftalmol. (Engl. Ed.).

[17] Hayashi K., Manabe S., Hirata A., Yoshimura K. (2020). Posterior vitreous detachment in highly myopic patients. Investig. Ophthalmol. Vis. Sci..

[18] Ramovecchi P., Salati C., Zeppieri M. (2021). Spontaneous posterior vitreous detachment: A glance at the current literature. World J. Exp. Med..

[19] Jonas J.B., Spaide R.F., Ostrin L.A., Logan N.S., Flitcroft I., Panda-Jonas S. (2023). IMI—Nonpathological Human Ocular Tissue Changes with Axial Myopia. Investig. Ophthalmol. Vis. Sci..

[20] Li S.T., You E.P., Wong A.H., Yeung J.C., Yu L.W. (2017). Management of traumatic haemorrhage in the Berger’s space of a 4-year-old child. Int. Ophthalmol..

[21] Nagarajaiah S., Shun-Shin G.A. (2011). Pigment deposition on the central aspect of the posterior lens capsule in pigmentary dispersion. Digit. J. Ophthalmol..

[22] Al-Mezaine H.S. (2010). Central posterior capsule pigmentation in a patient with pigment dispersion and previous ocular trauma: A case report. Indian J. Ophthalmol..

[23] Salman A., Parmar P., Coimbatore V.G., Meenakshisunderam R., Christdas N.J. (2009). Entrapment of intravitreal triamcinolone behind the crystalline lens. Indian J. Ophthalmol..

[24] Dubrulle P., Fajnkuchen F., Qu L., Giocanti-Aurégan A. (2016). Dexamethasone implant confined in Berger’s space. Springerplus.

